# Obesity, a major risk factor for immunity and severe outcomes of COVID-19

**DOI:** 10.1042/BSR20210979

**Published:** 2021-08-20

**Authors:** Mohammad Tohidul Amin, Kaniz Fatema, Sayema Arefin, Fahad Hussain, Dipty Rani Bhowmik, Mohammad Salim Hossain

**Affiliations:** 1 Department of Pharmacy, Noakhali Science and Technology University, Noakhali-3814, Bangladesh; 2 Department of Applied Chemistry and Chemical Engineering, Noakhali Science and Technology University, Noakhlai-3814, Bangladesh; 3 Department of Pharmacy, Mawlana Bhashani Science and Technology University, Santosh, Tangail-1902, Bangladesh

**Keywords:** ACE-2, COVID-19, Immunity, Morbidity, Obesity, SARS-CoV-2

## Abstract

An influenza-like virus named severe acute respiratory syndrome coronavirus-2 (SARS-CoV-2) is responsible for COVID-19 disease and spread worldwide within a short time. COVID-19 has now become a significant concern for public health. Obesity is highly prevalent worldwide and is considered a risk factor for impairing the adaptive immune system. Although diabetes, hypertension, cardiovascular disease (CVD), and renal failure are considered the risk factors for COVID-19, obesity is not yet well-considered. The present study approaches establishing a systemic association between the prevalence of obesity and its impact on immunity concerning the severe outcomes of COVID-19 utilizing existing knowledge. Overall study outcomes documented the worldwide prevalence of obesity, its effects on immunity, and a possible underlying mechanism covering obesity-related risk pathways for the severe outcomes of COVID-19. Overall understanding from the present study is that being an immune system impairing factor, the role of obesity in the severe outcomes of COVID-19 is worthy.

## Introduction

Pandemic severe acute respiratory syndrome coronavirus-2 (SARS-CoV-2), first identified in Wuhan, China, is responsible for COVID-19 [1,2]. Millions of individuals have been infected by this virus and has caused the deaths of more than 4.2 million people worldwide up to July 2021 [3]. The genomic characterization of SARS-CoV-2 was first done on 7 January 2020, and it has a specific difference in structure than previously identified pandemic virus SARS-CoV and Middle East respiratory syndrome-coronavirus (MERS-CoV), though they are closely related [4]. Initially, it was thought that COVID-19 is less complicated than SARS-CoV and MERS-CoV. However, in the recent case of illness, the rapid transmission level from human to human and the percentage of mortality worldwide implies that SARS-CoV-2 is more severe than previously identified SARS-CoV and MERS-CoV [4,5]. World Health Organization recommends dry cough, dyspnea, fever, sputum production, headache, bloody cough, diarrhea, lymphocytopenia, fatigue as symptoms of COVID-19 disease. Symptoms of viral pneumonia observed in severe cases may lead to acute respiratory distress syndrome (ARDS), acute cardiac injury, the incidence of grand-glass opacities, and finally, death [6,7]. Patients who are currently suffering from obesity (BMI ≥ 35) or overweight, hypertension, diabetes, and cardiovascular diseases (CVDs) are very susceptible to severe infection and have a higher risk of morbidity if infected by SARS-CoV-2 [1,4,8–10].

In the current world, obesity is increasing highly with a prevalence of one-third of the total population, and the rate is very high in U.S.A. (42.4%) and Europe (53.1%) [11–13]. In pulmonary infection, obesity is described as an independent risk factor [14]. Increased abdominal fat hinders the ventilation of the lung base, resulting in increased oxygen demand in the blood. [15]. The incidence of abdominal obesity is high in the Asian population. Abnormal secretion of cytokines like interferon, tumor necrosis factor-α (TNF-α) from abdominal adipose tissue is responsible for chronic low-grade inflammation. Such inflammation suppresses the immune system and impairs lung function [16]. Individuals who are obese face a very high risk of severe consequences in COVID-19, as obesity is a risk factor for several chronic disease conditions [17,18]. A French study indicates that 47.6% of patients with obesity (BMI > 30 kg/m^2^) and 28.2% of patients with severe obesity (BMI > 35 kg/m^2^) were admitted to the hospital to manage COVID-19 [19]. In addition, the percentage of the patients who require invasive mechanical ventilation is more for increasing body mass index category [19]. Furthermore, obesity is correlated with multiple disease conditions like hypertension, diabetes, and cardiovascular illness, responsible for morbidity worldwide [20,21]. In particular, hypertension, diabetes, and CVD are more significant risk factors for COVID-19 morbidity [22]. A very high mortality rate for patients with obesity in pandemic influenza 2009 showed the course of interaction between obesity and infection with the H1N1 influenza virus [16,23–29].

The pandemic COVID-19 has rapidly spread across international borders of 222 countries, and a very high spread is observed in Europe, North America, Australia, and Asia, where obesity is highly prevalent. Up to 28 July 2021, almost 35487490 cases and 628098 deaths were confirmed in the United States, and 33956561 COVID-19 positive patients and 742847 deaths were officially reported in the European Union and the United Kingdom [30,31]. India, an Asian country, is similarly plagued by a high prevalence of infection and death by COVID-19 [3].

Both obesity and COVID-19 disease have a very high global incidence, and clinical evidence indicates that obesity is associated with severe COVID-19 disease outcomes. However, the correlation between COVID-19 disease’s severe outcomes and obesity is not officially established. Therefore, it is critical to close this gap immediately, as the death rate has been steadily increasing since the outbreak began. The current study aims to determine the global prevalence of obesity and COVID-19 disease and develop a coherent association among obesity, immunity, and COVID-19.

## Prevalence of obesity and COVID-19

One of the most public health problems on the planet is obesity. It is positively associated with other health conditions such as hypertension, CVD, diabetes, end-stage renal disease, and to some extent, cancer [32–38]. The prevalence of worldwide obesity is very high. Currently, one-third of the world’s total population is obese or overweight. The rate of obesity has doubled within 40 years [11]. The overall summary of the epidemiological distribution of obesity is represented in Table 1.

**Table 1:** Prevalence of worldwide obesity

Geographical region		Percentage of obese people	Resources
North America	U.S.A.	Average obesity 42.4% Severe obesity 9.2% Non-Hispanic black 49.6%	[39]
	Canada	38%	[40]
South America	Brazil	General obesity 20.7% Abdominal obesity 38%	[41–43]
	Native Americans	72%	[44]
Europe	Overall	53.1%	[13]
	Switzerland	43.3%	
	Denmark	45.2%	
	Belgium	46.8%	
	Sweden	50.0%	
	Netherlands	50.1%	
	Austria	50.8%	
	Ireland	50.8%	
	Norway	51.5%	
	Poland	53.6%	
	Spain	53.8%	
	Germany	54.9%	
	United Kingdom	54.9%	
	Estonia	55.2%	
	Finland	55.5%	
	Portugal	57.1%	
	Slovenia	58.0%	
	Lithuania	59.6%	
	Czech Republic	60.1%	
	Hungary	61.6%	
Asia	China	General obesity 13.2% Abdominal obesity 44%	[45–47]
	South Asia	Varied range of abdominal obesity	[48–51]
	Iran	21.7%	[52]
	Saudi Arabia	27.6%	[53,54]
	Qatar	40.4%	[55]
	Kuwait	55.3%	[55]
Africa	Native African	1.3–47.7%	[56–58]
	Immigrant Africans	3.6–49.4%	[56–58]
Oceania	Australia	Men: 27.5% Women: 29.8%	[59]
	New Zealand	Men: 28.1% Women: 30%	[60]

Pandemic disease causd by SARS-CoV-2 or new coronavirus has spread rapidly across the world. After the first detection at the end of December 2019 in Wuhan, China, it spread to almost 222 countries up to July 2021 [1,2,61]. The virus spread epidemically in the United States, Europe, China, South Asia, Latin America, and Africa. The number of confirmed cases exceed 186 million, and the number of deaths worldwide due to COVID-19 disease is more than 4 million. Published research found that obese people are more likely to be infected with respiratory viruses and encounter a greater degree of illness and negative impacts, including higher infection rates, ICU, and death [62,63]. A higher incidence of obesity (41.7%) was observed in the case studies of 5700 patients hospitalized with COVID-19 disease in New York City, indicating obesity as an understated risk factor for severe outcomes of COVID-19 [64]. Intensive Care National Audit and Research Center (ICNARC) report stated that the patients admitted to the ICU for COVID-19 related complications in the United Kingdom are 38% obese [65]. Data from China also found that obesity raises the risk of extreme COVID-19 almost three-times as long as hospital stay raises [66]. The French retrospective analysis on ICU admitted COVID-19 patients found that 76% of patients were overweight [19]. So worldwide, very high prevalence of obesity makes the COVID-19 patient highly susceptible to higher disease complications.

## Impact of obesity on immunity

Obesity is associated with a metabolic disturbance with a high risk for some other chronic disease [67,68]. Obesity-induced dysfunctions of the immune system are responsible for the progression of some chronic diseases and metabolic impairment. Obesity-related physiological dysfunction causes fat accumulation in the lymphoid tissue that ultimately breaks the tissue structure and integrity of lymphoid organs, disturbing the leukocyte population and lymphocyte function [69,70]. The bone marrow-derived pluripotent hematopoietic stem cell is responsible for producing lymphoid and myeloid type blood cells. NK cell, B and T lymphocytes are lymphoid types, macrophage, monocyte, granulocyte, erythrocyte, megakaryocyte, dendritic cell, and mast cells are myeloid type cells. In the further development of T lymphocyte, the thymus plays a significant role [71–75].

Obesity causes the deposition of fatty content in the lymphoid tissue, changes tissue architecture, and increases the lymphoid tissue’s inflammatory gene expression [72,76]. These ultimately affect hematopoietic niches and suppress the erythropoiesis process [77,78]. In addition, obesity is strongly linked with the alteration of thymic tissue structure, which is correlated with increasing age [72,79]. The obesity-induced change in thymic architecture is responsible for the lower thymic output of naive T cells and ultimately reduced immune function [72,80]. T-cell infiltration in adipose tissue is observed high in individuals with obesity and, to some degree, the lymphocyte activation antigen produced from obese adipose tissue [81]. Again B-cell activity is also regulated in response to high-fat diet-induced obesity [82]. An overall summary of the effect of obesity on the immune system parameter is presented in Table 2.

**Table 2:** Impact of obesity on the immune system

Parameter of the immune system	Model	Level of change in the immune system	Resources
Development of leukocyte	HFD-fed mice	↑Myeloid progenitor cells ↓Lymphoid progenitors ↓Thymic output of naive T cells	[72]
	Obese and insulin-resistant patients	↓Thymic output of naive T cells	[72]
	Adipocyte-rich bone marrow in C57BL/6J mice	↓ Hematopoiesis	[77]
	Leptin receptor-deficient mice	↓ Hematopoiesis	[83]
Inflammation of leukocytes	HFD-induced obese mice	↑ T-cell infiltration in adipose	[84]
		↑M_1_ macrophages	
		↓ M_2_ macrophages in adipose	[85]
		↑ T_H_1 ↓ T_reg_ cells in adipose	[81,86]
	Obese human subjects	↑ CD4^+^ T cells ↓ CD8^+^ peripheral T cells	[87]
		↑ NF-κβ activation in PBMCs	[88]
		↑Migration inhibition factor (MIF), IL-6 ↑ TNF-α ↑ MMP-9 mRNA expression in PBMCs	[88]
	Morbidly obese human subjects	↑ T_H_1 and ↑ T_reg_ cells	[89]
		↑ CD4^+^ and CD8^+^ T-cell proliferation	[89]
Lymph	HFD-induced obese mice	↓ Inguinal lymph node size ↓ T-cell count	[90]
		Impaired lymphatic fluid transport, and dendritic cell migration	[90]
Bone marrow	Obese male and female	Adiposity in bone marrow	[91]
	HFD-fed Wistar rat	↑ Proinflammatory gene expression of mesenchymal stem cells	[92]
Spleen↑	HFD-fed mice	Increased memory T cell ↓ T-cell receptor diversity	[72]
Thymus	HFD-fed mice	↑ Thymic involution and adiposity	[72]
	Leptin deficient (ob/ob mice)	↓ Thymus size and cellularity ↑ Thymocyte apoptosis	[93]
Clinical leukocyte profiles	Weight loss, overweight, and obese subjects	↓WBC count	[94]
Immunity parameters	Diet-induced obese mice	↓ Dendritic cell antigen presentation ↓ Maintenance of influenza-specific CD8^+^ memory T cells	[95]
	Leptin-deficient (ob/ob) mice	↓ Cell-mediated immunity	[96]
	Diabetic and obese mice	↑ Lung cancer metastasis ↓ NK cell function at early cancer stages	[97]
	Overweight children and child obesity	↑ Tetanus vaccine failure risk	[98,99]
	Obese adults	↑ Risk of influenza vaccine failure ↑ Allergic disease	[100]
	High obesity prevalent in community	↑ Influenza-related hospitalizations	[101]
Inflammatory cytokines and chemokines	Diet-induced obese mice	↓ TGF_β_ concentration in the lung and ↑ TGF_β_ concentration in BALF	[102,103]
		↑ TNFα concentration in plasma ↑ mRNA expression in lung ↑ BALF concentration	[103,104]
		↑ G-CSF concentration in lung	[105]
		↓ MIP1α concentration in BALF ↑ MIP1α concentration in lung	[105]
		↓ IL-5 concentration in BALF at the time of infection	[105]
		↑ Leptin concentration in serum ↑ mRNA for Leptin expression in lung	[106,107]
		↓ IL-1β concentration in the lung and ↓ mRNA for IL-1β expression in the lung during influenza infection	[104,105,107]
		↓ mRNA for IL-2 expression in lung	[108]
		↑ IL-6 concentration in serum and lung during infection	[105,108]
		↓ Adiponectin concentration in serum and lung BALF	[103,107]
		↑ Plasma MIP2α concentration	[109]
		↑ MCP-1 concentration in BALF during infection	[110]
		↓ Lung mRNA expression for IFNα and IFNβ	[108]
Immune cell	HFD-induced obese mice	↓ Macrophage migration to the lung ↑ M1 polarization	[110]
		∼ NK cells count in lung	[102,108,110]
	Leptin-deficient OB model mice	↑ Number of alveolar macrophages in BALF	[110,111]
		↑ NK cells count in lung	[102,108,110]
	HFD-induced obese mice	↓ Plasmacytoid dendritic cell count in the lung ↓ Antigen presentation during influenza infection ↓ T-cell proliferation ↓ Lung double negative dendritic cell ↓ pDCs count during infection	[95]
		↑ Neutrophil polarization ↑ Neutrophil net production ↑ BALF infiltration during influenza infection	
		↓ Mature bone marrow B cells and cross-reactive H1N1 and PR8 antibodies during influenza infection	[112]
		↓ T-cell count ↑ OCR: ECAR ratios	[112,113]

Here, ↑, increased; ↓, decreased; ∼, unchanged. Abbreviations: BALF, bronchoalveolar lavage fluid; G-CSF, granulocyte-colony stimulating factor; HFD, high-fat diet; IL-2, interleukin-2; IL-5, interleukin-5; IL-6, interleukin-6; MIP1α, macrophage inflammatory protein 1α; MIP2α, macrophage inflammatory protein 2α; MMP-9, matrix metalloproteinase-9; NF-κβ, nuclear factor κ B; NK, natural killer; PBMC, peripheral blood mononuclear cell; pDC, plasmacytoid dendritic cell; PR8, Puerto Rico 8; TGF_β_, transforming growth factor-β; T_H_1, T-helper cell-1; T_reg_, regulatory T cell; WBC, white blood cell.

Chronic inflammation is a typical characteristic of obesity primarily due to weight gain and adipose tissue dysfunction caused by metabolic tissue stress. Hypertrophic adipocytes are more likely to trigger endoplasmic reticulum and mitochondrial stress, supporting chronic, proinflammatory activation in adipose tissue. These ultimately lead to inflammatory leukocyte infiltration and enhanced cytokine secretion [114,115]. Adipose tissue also secretes into circulation several proinflammatory cytokines, chemokines, and adipokines, contributing to low-grade chronic inflammation. Besides, viruses may also demonstrate tropism for various tissues and cell types like adipose tissues and adipocytes [116]. SARS-CoV-2 may also have adipose tissue tropism, leading to intrapulmonary and systemic inflammation [117]. Chronic inflammation associated with obesity can inhibit macrophage activation and relocation, disrupt the formation of neutralizing antibody and memory T cells, and decrease activation of the immune system’s effector cells, suppress immune functions and host defenses [118,119]. Thus, we conclude that chronic inflammation of obesity, both systemic and local, leads to immune dysfunction, which increases the risk associated with severe outcomes of COVID-19 disease.

## Impact of obesity on influenza

A very highly contagious disease, influenza, that affects the respiratory system is caused by the influenza virus. It is highly contagious and responsible for affecting 3–5 million individuals and kills 290000–650000 people globally each year [120]. Only in the United States, ∼12000–56000 people die every year from the influenza virus [121]. Influenza virus is an RNA virus, and a lipid layer encapsulates the nucleic acid segment with two surface proteins, hemagglutinin, and neuraminidase. Four strains (Influenza Virus - A, B, C, and D) have been identified [122–124].

The Spanish flu pandemic in 1918 was the most devastating influenza virus attack in the last century. This virus attacked almost one-third of the world’s population, and ∼50 million people died worldwide [125–127]. No other influenza pandemic after the Spanish flu pandemic is so severe as the 1918 pandemic. Another serious influenza pandemic is *H*_1_*N*_1_ influenza, also called swine flu, spread in 2009 [128,129]. The virus spread rapidly in ∼168 countries, and approximately more than 123000 people died worldwide in 2009 [130]. Obesity was identified as an independent risk factor for higher morbidity resulting from *H*_1_*N*_1_ infection [131]. The previous record suggested that 61% of adult obese people died due to the influenza attack in 2009 [131]. Generally, obese people with BMI in the range of 30–35 kg/m^2^ have 1.45-times, and BMI greater than 35 kg/m^2^ have a 2.12-times higher risk of hospitalization in seasonal influenza [132]. The current pandemic COVID-19 is also an influenza-like disease and represented similar complications in the case of the overweight and obese patient.

## Impact of obesity on cardiovascular function

Obesity is a proven contributing factor for other lifestyle disorders like CVD, hypertension, coronary artery disease, diabetes mellitus (DM), insulin resistance (IR), renal dysfunction. Obesity mediated most significant change in morphology of cardiac system, that is hypertrophy of left ventricle (LV), whereby high blood pressure and IR are essential factors for LV mass [133]. Obesity is also involved with heart failure and diastolic dysfunction of LV [134]. Obesity amplifies the effects of multiple cardiovascular risk factors, accelerates the development of CVD, and has a detrimental impact on cardiorenal function. As part of this, an adverse effect on the myocardium occurred due to obesity-mediated activation of the renin–angiotensin–aldosterone system (RAAS), resulting in overexpression of angiotensin II [135]. In addition, obesity is also associated with an increased risk of thrombosis. This is due to chronic inflammation caused by obesity, which results in the down-regulation of anticoagulant regulatory proteins (antithrombin, protein-C, and TFPI), the overexpression of coagulant factor (tissue factor), and adhesion molecule (P-selectin), all of which increase thrombin synthesis, platelet activation, and ultimately thrombosis [136]. Again an individual with obesity has some metabolic considerations like reduction in β-cell function and development of IR. As a result, it is difficult for an obese individual to cope up metabolically and immunologically with a severe infection like COVID-19. Altogether, the integrated metabolic control needed for complicated cell interactions and efficient host protection is disrupted, causing a functional immunological deficiency.

In the destruction of pancreatic β-cells, SARS-CoV-2 plays a role through interactions with angiotensin-converting enzyme-2 (ACE-2). Again, in patients with COVID-19, acute cardiac injury is particularly prominent and is correlated with severe clinical outcomes [4]. However, all the heart failure cases (23%) noticed in hospitalized COVID-19 patients were not pre-existing cardiomyopathy [137]. In a study of 150 COVID-19 patients, the definitive cause of death in 7% of patients was acute myocarditis [138]. However, histology of the postmortem myocardium demonstrated rapidly progressive myocarditis with inflammatory mononuclear infiltrates in the myocardial tissue [139]. These findings suggest a correlation between obesity and COVID-19-mediated acute cardiac injury and related severe outcomes.

## Obesity and its impact on lung function

The very high prevalence of obesity increased the risk of morbidity and clinical feature of many respiratory diseases, as it causes a significant change in lung and chest wall function. These functional and mechanical changes in the lung and airway wall cause asthma, dyspnea, obstructive sleep apnea, obesity hypoventilation syndrome, airway hyperresponsiveness, wheeze, ARDS, chronic obstructive pulmonary disease (COPD), and pulmonary hypertension [15,140,141]. In obese subjects, the deposition of fat in the mediastinum and abdominal cavities alters the lung and chest wall’s mechanical properties, thus changes the lung’s structure, physiology, and function [15,142,143]. This changed structure also limits the breathing pattern. Generally, air flows into the lung due to the negative pressure gradient within the pleural space. As fat deposits in the thoracic and abdominal area, pleural pressure and intra-abdominal pressure increased in obese subjects, ultimately restricting the diaphragm’s downward movement and outward movement of the chest wall [144,145]. These results reduce functional residual capacity (FRC), proportional to obesity from overweight to severe obesity [146]. Tidal volume is also slightly reduced, and a shallow breathing pattern is noticed, increasing overall minute ventilation [147,148]. Moreover, the mechanical effect of obesity causes narrowing of the airway, which leads to gas tapping, respiratory inhomogeneity, and resistance [149].

Excessive adipose tissue elevation is associated with increased inflammatory cytokines and the immune cell, causing lung function disturbance [15]. In obese individuals, the expression of proinflammatory adipokine leptin increases that plays a role in the ventilator drive and worsens the asthmatic conditions [150,151]. Other inflammatory chemokines like TNF-α, interleukin-6, interleukin-8, monocyte chemoattractant protein-1, and high sensitivity C-reactive proteins (hs-CRPs) increased in obesity [152,153]. The development of obesity also increases macrophage infiltration and mast cell propagation [151,154]. Mast cells are critical mediators for an allergic reaction, and obesity-induced mast cell proliferation is a potential mechanism of airway dysfunction. Increased levels of circulating leukocytes have been documented in obesity [155]. There is strong evidence that airway disease and lung dysfunction are associated with chronic inflammation during adipogenesis [154–156].

## Pathway of COVID-19 infection

SARS-CoV-2 is transmitted by zoonotic transmission from animal to human and spread rapidly among humans via respiratory droplets and fecal–oral transmission. The symptoms developed within 11.5 days on average, and symptoms are dry cough, fever, muscle pain, joint pain, difficult breathing, diarrhea, dizziness, headache, nausea, and blood coughing [4,157,158]. Previous studies on SARS-CoV suggested that this virus principally targets the airway epithelial cell layer, vascular endothelial cell, alveolar epithelial cell, and macrophages in the lung, expressing a protein called ACE-2 [159,160]. ACE-2 is the target receptor for SARS-CoV-2 [161]. In obese individuals, more significant adipose tissue proliferation contains adipocytes that have higher expression of ACE-2 receptors. The ACE-2 receptor count of the fatty tissue in obese individuals is much higher than lung, which makes fat a potential reservoir for SARS-CoV-2 as it is the entry site of the virus [162].

**Figure 1:**
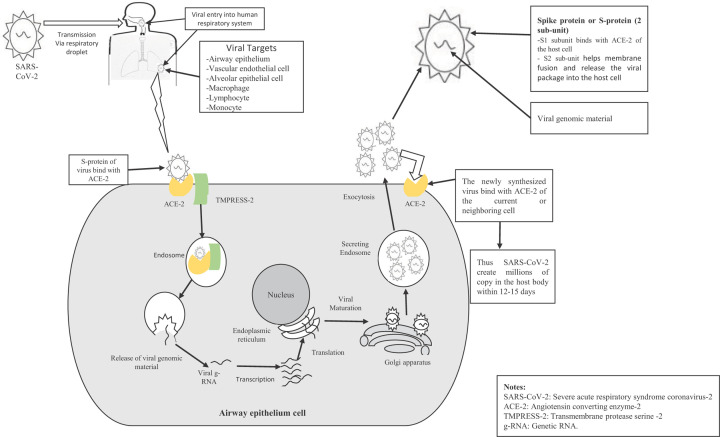
Typical mechanism of SARS-CoV-2 viral transmission and replication in the airway epithelium cell

The coronavirus has a large homogeneous protein called Spike protein or S protein, which gives the viral characteristic crown-like appearance. S proteins have two subunits: the S1 and the S2. The receptor-binding domain in the S1 subunit binds with ACE-2 of the host epithelial cell, triggering a sequence of events that enter the SARS-Cov-2 virion into the host cell [163,164]. The S2 subunit consists of two heptad repeat regions (HR-1 and HR-2) and a fusion peptide region (FP). The transmembrane protease serine-2 (TMPRSS-2) plays a role in triggering the cleavage of S protein of SARS-CoV-2 and releasing viral genetic material into the host cell. The overall mechanism of viral transmission and replication in the host cell is represented in Figure 1.

Within the endosome, the S1 subunit cleaved away. The S2 subunit folds by itself, thus bringing the HR-1 and HR-2 regions together that helps in membrane fusion and release the viral package into the host cell cytoplasm [165–167]. Coronavirus genetic material is a single-stranded RNA that can replicate by using replicating material from the host cell. The virus uses the host cell ribosome to replicate polyprotein [168]. Like the main coronavirus proteinase (3CLpro) and the papain-like protease (PLpro), the two enzymes are involved in expressed polyprotein cleavage, and the cleaved product is used for replicating a new virus. An RNA-dependent RNA polymerase is expressed by coronavirus to synthesize the daughter RNA genome, synthesizing the complementary RNA strand using virus RNA as a template [169]. Thus the virus produces millions of copies in the host body. The SARS-CoV-2 virus is cytopathic responsible for injury or death of virus-infected cells and tissue-associated vascular leakage as part of the virus multiplication cycle [170–172]. The cytopathic virus is responsible for programmed cell death by pyroptosis and subsequent inflammatory response [173,174]. The clinical study suggested that COVID-19 patients admitted in the hospital and required intensive care have elevated plasma levels of TNF-α, IL-2, IL-6, IL- 10, G-CSF, IP-10, MCP-1, and MIP-1α [4]. Under COVID-19 disease condition, this elevated plasma level of different inflammatory mediators accelerates the chronic inflammatory condition due to obesity. Thus, the virus SARS-CoV-2 activates macrophages, monocytes, and B cells in the local immune response and kills the lung cells. A dysfunctional immune response is also noticed in some cases, which causes severe lung and systemic pathology.

## Obesity and COVID -19 pandemic

The pandemic COVID-19 has already shown its gruesomeness worldwide, causing a vast number of human deaths. The infection of COVID-19 becomes complicated with pre-existing comorbidity like diabetes, CVD, renal failure, and others. Recently, WHO declared obesity as an independent risk factor for case severity in COVID-19 disease [15]. Italian National Institute of Health launched a study on COVID-19 throughout the country and reported that pre-existing non-communicable diseases like obesity, diabetics, hypertension CVD, and renal failure are responsible for 99% of COVID-19-related deaths [175]. A study in New York suggested that younger patients (age < 50) with a BMI above 40 kg/m^2^ is a risk factor for COVID-19 mortality [176]. U.K. Intensive Care National Audit and Research Centre reported that two-third of the total people with serious COVID-19 complications were overweight or obese according to the WHO obesity scale [177]. Another study in the United States shares their experience that people having obesity aged less than 60 years is a newly identified epidemiologic risk factor for COVID-19 morbidity [178].

**Figure 2:**
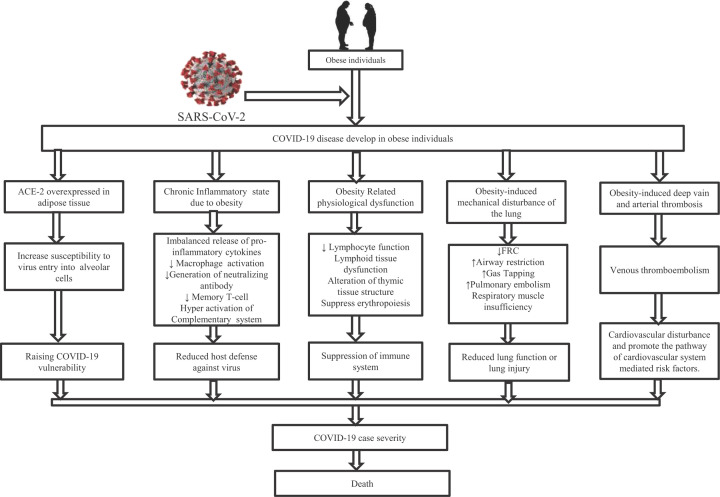
Possible biomolecular pathways through which obesity promotes the severe outcomes and mortality of COVID-19 disease condition

Several molecular events can explain the correlations between obesity and severe outcomes of COVID- 19, as displayed in Figure 2. How ACE-2 plays a role in the endocytosis of SARS-CoV-2 is clearly described in the Pathway of COVID-19 infection section. Generally, ACE-2 is overexpressed in adipocytes; thus, the elevated level of ACE-2 in obesity might play a role in cross-talking between obesity and COVID-19 case severity [162,179–181]. We presumed that obesity-induced ACE-2 overexpression, as a functional receptor for SARS-CoV-2 invasion, may play a role in acute respiratory failure progression can be a factor in increasing COVID-19 vulnerability. In another way, obesity alters the immune function causing an imbalanced release of inflammatory cytokines that weaken the host defense against influenza type virus [182–184]. Chronic inflammatory state due to obesity is the responsible factor for the imbalanced release of proinflammatory cytokines, inhibition of macrophage migration and activation, impair the formation of neutralizing antibody and memory T cells that suppress the immune system activation, and host defense against SARS-CoV-2 [126–128].

It is interesting to note that excess fat is associated with the complementary system’s hyperactivation, theoretically capable of inducing inflammatory sequelae, eventually developing a condition described as ‘cytokine storm’ [185]. These may help disrupt lung function during SARS-CoV-2 infection. The previous study suggested that obese individuals also have a risk of vaccination failure [109]. Again, seriously infected patients with COVID-19 and obesity under intensive ventilation due to reduced oxygen saturation levels are difficult to regain normal oxygen saturation due to obesity-induced mechanical dysfunction of the lung [182]. General obesity and abdominal obesity are responsible for increasing airway resistance, reduced FRC, respiratory homogeneity, respiratory muscle insufficiency, increased pulmonary embolism, and gas tapping. These ultimately cause the reduction in overall lung function or lung injury. This type of lung functioning hindrance causes case severity and increased morbidity in individuals with obesity. Obesity-induced physiological dysfunction of the immune system cell also promotes the overall suppression of immunity for defense against SARS-CoV-2. A recent study on 247 COVID-19 patients demonstrated that the rate of hospitalization reduced with higher cardiorespiratory fitness found low in obese individuals [186,187]. In another way, obesity enhances thrombosis and venous thromboembolism that open the pathway of cardiovascular disturbance related to COVID-19 risk factors.

## Conclusion

COVID-19, a kind of influenza virus, is highly contagious, spread worldwide quickly, and declared a pandemic by the WHO. At the same time, obesity is highly prevalent worldwide and has been documented for impairing the adaptive immune system and responsible for mechanical dysfunction of the lung. Thus, including the previously established risk factors like diabetes, hypertension, CVD, renal disorder, obesity must be enlisted as individual risk factors for severe COVID-19 and mortality outcomes. Understanding the underlying mechanism of obesity-mediated severe outcomes of COVID-19, a diet enriched with the immune system booster and regular physical exercise can be a preventive measure for obese individuals to reduce the risk of the severe outcomes of COVID-19. Traditional health campaigns conveying the information of obesity-mediated case complications of COVID-19 may raise awareness among the general population. Further study with clinical research using the underlying mechanism may promote newer treatment options for obese COVID-19 patients.

## Abbreviations

ACE-2: Angiotensin-converting enzyme-2
ARDS: Acute respiratory distress syndrome
BMI: Body mass index
COPD: Chronic obstructive pulmonary disease
CVD: Cardiovascular disease
FRC: Functional residual capacity
IR: Insulin resistance
LV: Left ventricle
MERS-CoV: Middle East respiratory syndrome-coronavirus
SARS-CoV-2: Severe acute respiratory syndrome coronavirus-2
TNF-α: Tumor necrosis factor-α
TMPRSS-2: Transmembrane protease serine-2
